# Opening the conversation in peer review, finally

**DOI:** 10.1590/0074-02760250005

**Published:** 2025-09-05

**Authors:** Adeilton Alves Brandão, Ana Carolina P Vicente

**Affiliations:** 1 Fundação Oswaldo Cruz-Fiocruz, Instituto Oswaldo Cruz, Rio de Janeiro, RJ, Brasil

As we always do when April of each year comes around, we are again thinking about the celebrations of Memórias do Instituto Oswaldo Cruz anniversary. Of course, we tend to celebrate more enthusiastically the counting of years that ends in “0” or “5” digits, *e. g.*, 50, 100, 115. Such numbers might lead to a strong commitment to celebrate relevant and historical achievements, yet in April 2025 Memórias do IOC will not celebrate an anniversary that ends in 0 or 5 digits. We are right now celebrating 116 years of publishing the first edition of this journal (officially Memórias do IOC was created on December 12th 1907, but the first print edition appeared in April 1909).^1^ It does not mean however that an anniversary mark ending in digits other than zero or five is not worth celebrating. Rather, we consider this event quite an opportunity to make important announcements. And here is the message that comes along with Memórias do IOC 116th anniversary: starting in April 2025, this journal will publish along with the main article all the conversation between editor, reviewers and authors. Memórias do IOC will follow suit what other scientific journals have been doing in the last years: open the peer review and strive to get the highest alignment to open science. Since our December 2017 announcement in support of the open science and preprints initiative, this is a long awaited decision.^2^

The moment for such a decision is also an opportunity to look at (and think about) the procedures Memórias do IOC has been deploying to handle the manuscripts it has been receiving since the first edition in 1909. Throughout nine decades the editors of Memórias do IOC used a procedure for register and control of the submitted manuscripts that was entirely based on “notebook entries”. The “notebook procedure” generated several volumes that recorded the registration and handling of the received articles. Unfortunately not all of the physical volumes have been preserved. The oldest one dates from the year of 1928. We may read therein the day and month each article was submitted and how it was handled. It is not clear however whether these manuscripts were forwarded to external peer review. As evaluation of manuscripts by external peers was not a standard practice for editorial decision at that time, most probably they were not forwarded to peer review. The “notebook” registration procedure lasted until the end of the 1990 decade. At the dawn of 21st century Memórias do IOC ceased to use such a procedure and started using digital submission systems. Initially it was carried out through electronic mail, and later on by using specific software for ‘manuscript submission and handling”, such as the Open Journal System, and currently the proprietary software ScholarOne.

External peer review in Memórias do IOC started around 1980 but again, the internal records of the journal do not give details about it. That year also witnessed the extensive reorganisation of the journal that happened under the leadership of Prof José Rodrigues Coura, who was simultaneously the Director of Instituto Oswaldo Cruz and Editor-in-Chief of Memórias do IOC. The ensuing steps defined a turning point for Memórias: the transformation from a mostly institutional journal to an international one in the field of parasitology and tropical medicine. That was a major change both in scope and operations that took more than a decade to complete (for example, the adoption of English as the journal official language occurred nine years later, in 1989). Indeed, we think it is still a “work in progress” as each year a new challenge is posed to us to keep moving in the “international arena” for proper dissemination of human infectious diseases research.

In the last 10 years we have been advocating for more transparency in editorial practice, less barriers to science dissemination, for actions that reduce the cost of scientific publication, for data opening and wide dissemination, in summary for a radical immersion in open science.^3^, ^4^, ^5^, ^6^, ^7^, ^8^, ^9^, ^10^,^-^ ^11^ So why didn’t we open the peer review earlier? Many motives could be listed to explain the delay in such a decision and yet none of them alone would suffice. They all add up and converge to a point relevant to academia: the uneasiness to abandon (or reform) consolidated practices (at least for the biomedical sciences community in this country). This is not only a question of culture or tradition, rather it is a discussion on incentives and pragmatic decisions towards a successful scientific career and personal prestige. The foremost driving force in science should be the searching for solutions to relevant problems that defy current human knowledge. In other words it means pushing the frontiers of science to both unpaved and unmapped routes. Regardless of being either “applied” or “pure” knowledge, that is, to know how to do/solve things/problems or simply getting new information about how nature works, a scientific effort should reward all the people involved in it for the sake of “having a relevant job done efficiently”.

That is what happens in an ideal world. In the pragmatic one we live in there are other factors that turn scientific venture into something that is boringly predictable and materially rewarding. It is just a game of who scores better in the amount of approved grants, published articles in high profile journals, of countless citations, mentions in the mass media, likes in social media, page views and downloads, invitations to speak in scientific meetings, guest editors of special issues, and of “prizes and awards”. But what is really worrying is that for each item above there are the respective scum and fraudulent schemes to deceive those who are concerned with and complying to “good publishing practice”. It does not matter if the journal sponsors and the meeting organisers are just interested in making easy money, if the shallowness of published research work did not deserve any further comment, the hypothesis and data analysis are simply flawed (or irrelevant in the best scenario) and the resources were far from efficiently used. All of those things and deeds are counted and elevated to “the best of our knowledge and efforts’’!

Science arena is now crowded with “good ones”, “bad ones”, “good things’, “worthless things”, “good habits” and “practices to be definitively avoided”. It seems more cumbersome than we thought it could be. A cynic might say that this science game is a typical human legacy (or product) and for every novelty or period in human history things were no different. There is a race for every endeavour human beings are engaged in, and the winners and losers of this race will depend on their skills to get the most favourable balance of the rewards and drawbacks. As a reminder of the inexorability of this race there are the ubiquitous lists of “most cited”, “most productive”, “most funded”, “most praised”, “most awarded”, “who influences the most”, “who is who” and so on that routinely lead us to believe that these people are making the humanity better than it actually is. No, we are not getting better in our human endeavours despite the massive amount of scientific research published every single day.

Memórias do IOC is a kind of secular witness to the impressive increase in research output. Since its beginning Memórias has published more than 7.000 articles, which are mere sand grains compared to the current global scientific output. As a journal that started its journey as a vehicle to disseminate (mostly but not exclusively) what the professionals at Instituto Oswaldo Cruz were researching inside its walls, this number says a lot about the productivity of individual scientists in the beginning of both 20th and 21st century. On average scientists now appear to publish a lot more than their counterparts at the start of the 20th century. It is easy to verify this statement but before extracting the figures from the scientific database we need to think deeply about two disturbing questions:

(a) Are the current scientists more creative and innovative than the century past peers?

(b) Are we really in need of such a vast amount of “fresh knowledge” published every day?

The gamers of science certainly have an answer to these questions…

For the time being and on account of well-balanced use of scarce resources we only wish that the overwhelming (and to a large extent useless) scientific output drops from the current peak.
